# Preface

**DOI:** 10.3205/cto000149

**Published:** 2017-12-18

**Authors:** Dirk Eßer

**Affiliations:** 1 Hals-Nasen-Ohrenklinik, Helios Kliniken, Erfurt, Germany

## Preface

Dear Colleagues,

in the long history of the German Society of Oto-Rhino-Laryngology, Head & Neck Surgery, it was now for the second time that our annual meeting took place in Erfurt (May 24–27, 2017). Nine key note lectures and written contributions reflected the main topics dealing with diagnostics and therapy of hearing disorders as well as vertigo, tympanoplasty, idiopathic sudden hearing loss, the treatment of vestibular schwannomas, cochlear implantations, and the state-of-the-art of hearing aid provision.

At this point, I want to extend my special thanks to all the authors and co-authors of the articles. I am fully aware of how much work and effort was necessary to create “manuals” on specific topics in a highly effective way. You may all be very proud of your contributions.

Another focus of this year’s congress was the special program for medical assistants in ENT practices and nurses working in ENT departments and hospitals. Particular emphasis was placed on practice-related topics with independent application.

Furthermore, at our “days of the private practitioner”, we discussed in cooperation with and under the responsibility of the Professional Association of Otolaryngologists various topics which had been chosen as closely related to everyday issues of the private practitioner.

The high number of submitted abstracts showed how multifaceted our discipline is. It also showed the high relevance of the sensory organs we have to examine and treat. Hearing, smelling, tasting, and the preservation or restitution of the senses of balance and hearing are important aspects of private and professional life. ENT-specific oncology, traumatology, and dysphagia were further main topics of the meeting program.

From my personal point of view, this meeting also summarized the year of my presidency. On the one hand we further expanded our German Study Center for Otorhinolaryngology, Head & Neck Surgery together with the Professional Association of Otolaryngologists; on the other hand we achieved a close relationship between our scientific society and the Professional Association. Based on the identification of key topics we will work on the so-called Agenda 2025 together with the Professional Association during the next years. In this context, the 88^th^ Annual Meeting represented a first milestone. The main topics that are highly relevant for the future of our discipline were in the focus of numerous invited keynote lectures and round table discussions.

A high number of internationally renowned experts provided an excellent basis for specific discussions on the highest level.

I want to express my personal thanks to all authors of the scientific contributions. You all contributed to the great success of the 88^th^ Annual Meeting with regard to scientific and practical contents that became obvious in the high number of participants, the industrial exhibition, and finally in a lively scientific exchange and intensive discussions – and visits of the state capital of Thuringia.

Prof. Dr. med. Dirk Eßer 

President of the German Society of Oto-Rhino-Laryngology, Head & Neck Surgery

(Figure 1 (Fig. 1))

## Figures and Tables

**Figure 1:**
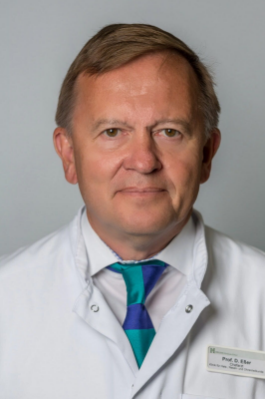
Prof. Dr. med. Dirk Eßer

